# Probabilistic empiricism

**DOI:** 10.1007/s13194-025-00653-5

**Published:** 2025-04-12

**Authors:** Quentin Ruyant, Mauricio Suárez

**Affiliations:** 1 Lancog Group, Centre of Philosophy, University of Lisbon, Lisbon, Portugal; 2 Interdisciplinary Complex Systems Group (GISC), Complutense University of Madrid, Madrid, Spain

**Keywords:** Empiricism, Probabilities, Modal epistemology, Situation semantics

## Abstract

Modal Empiricism in philosophy of science proposes to understand the possibility of modal knowledge from experience by replacing talk of possible worlds with talk of possible situations, which are coarse-grained, bounded and relative to background conditions. This allows for an induction towards objective necessity, assuming that actual situations are representative of possible ones. The main limitation of this epistemology is that it does not account for probabilistic knowledge. In this paper, we propose to extend Modal Empiricism to the probabilistic case, thus providing an inductivist epistemology for probabilistic knowledge. The key idea is that extreme probabilities, close to 1 and 0, serve as proxies for testing mild probabilities, using a principle of model combination.

## Introduction

Our past experiences seem to give us a great deal of modal knowledge: knowledge about what is objectively possible or not in this world, in various circumstances. We heavily rely on such knowledge in order to plan future actions. Yet the way this knowledge is acquired is prima facie puzzling: after all, all of our past experiences are located in the actual world, so how could we know anything about what happens in other possible worlds?

Ruyant (2021, ch. 3–5) has proposed a modal epistemology, called Modal Empiricism (ME),^1^ that aims at addressing this question without attempting to reduce or eliminate objective modalities, as empiricism traditionally does. The view is roughly the following: (1) we should think of objective possibilities in *situated* terms, that is, not in terms of possible worlds, but in terms of possible situations, conceived of as alternative ways local, coarse-grained situations could be in some relevant respects, “all else being equal”, and (2) if we do so, then necessity claims, in the form of claims about what holds for all possible situations of a given type, can be justified by simple enumerative induction, which is an “empiricist-friendly” form of induction. All that is required is to assume that instantiated possible situations are representative of merely possible ones.

Although concerned with modalities, ME does not say much about the status of probabilities, and on the way of justifying probability claims or the accuracy of probabilistic models. This is problematic, because the fact that many scientific models are probabilistic is one of the reasons to assume that there are objective possibilities in the world in the first place. Suárez (2020) has described the typical assumptions of statistical models in science, and the ensuing Complex Nexus of Chance (CNC), and our aim in this paper is to bring CNC to bear on ME, thus extending ME to the probabilistic case, and hence providing a coherent empiricist epistemology for objective probabilities. In Sect. 2, we review the main features of ME. In Sect. 3, we discuss various interpretations of probability, including the CNC and its compatibility with the spirit of ME. In Sect. 4, we explain how ME can be extended to the probabilistic case.

## Modal empiricism

### Experimental contexts and accuracy (ch. 3.2)

Let us start by reviewing the main features of ME.

In an empiricist spirit, ME is grounded in an account of model application, conceived of as the kind of representational activities where a model is used in order to make inferences about what is possible or not for a concrete actual situation that is accessed empirically. This is, supposedly, the kind of context capable of confirming or invalidating the empirical adequacy of the model, depending on whether the conclusions of the inferences that it affords turn out to be compatible or not with the observations made.

Following ME, a *context of application* defines what the applied model is about. A context is associated with a concrete physical system or situation under a given description (what is usually referred to as a *target* in the literature on scientific representation). It is understood in terms of a finite partition of logical possibility space for this situation: an exhaustive set of mutually exclusive coarse-grained a priori possibilities, one and exactly one of which will be realised, holding fixed the identifying characteristics of the situation of interest in all possibilities (including some environmental features).^2^ The situation, its identifying characteristics and the partition of possibility space considered (the coarse-graining) depend on the interests of model users. So, for example, if an agent uses a mechanical model in order to infer whether the ball she is about to drop will go up, down or stay at the same height, the context comprises exactly three mutually exclusive coarse-grained possibilities (up, down or same height) that cover the logical space of possibilities for the situation (dropping this ball), holding fixed the gravitational force, which ball is used, the absence of wind, etc.

Note that a context of application can be theory-laden. What matters is only that the possibilities delineated would make an empirical difference. In an experimental context where data is gathered in order to confirm or discard a hypothesis, the context could be the set of conceivable data models that could be obtained from the experiment, holding fixed the experimental setup (this is actually how Suppes (1969) conceived of models of the experiment). These data models would typically be described in the language of a theory, not in terms of instrument displays or the like. This reference to theoretical properties could seem to run against the spirit of empiricism, but according to Ruyant (2021, 3.4.2), all that matters is that experimental norms allow us to associate them with the interventions and observations of experimenters.

The general idea put forth by ME is that an applied model informs the user about which, among the logical possibilities delineated by the context, are *really* possible, in an objective sense of possible, thus reflecting the natural constraints that affect the identified situation. A model, if it is informative, will exclude some contextual possibilities, perhaps only leaving out a single one of them: in our illustration, a model of free fall would exclude every outcome but the one where the ball falls down, which is what must happen in virtue of natural constraints according to the model. An applied model is *accurate*, in a purely extensional sense, if what it excludes does not actually occur (the ball does not go up nor stay at the same height): the modal inferences that it affords are compatible with our observations of the situation.^3^

(Ruyant, 2021) briefly mentions the fact that applied models more typically attribute probability weights to contextual possibilities instead of simply excluding or allowing them, the latter being a limiting case of the former, and that the notion of accuracy should be refined in consequence. However, the way to do so is not elaborated. One of the aim of the next sections will precisely be to do so.

### Theoretical models and adequacy (ch. 3.3 and 4.2)

Theoretical models are not designed to be applicable only in a single context. A model of free fall, for instance, can account for a large range of situations where objects are dropped or thrown in various conditions, with various degrees of precision (various coarse-grainings). According to ME, the inferential content of such an abstract model should be analysed as a *function from context to content*. The idea is that given any situation with identifying characteristics, and given any partition of possibility space for this situation, the theoretical model can provide an objective weighing of the possibilities considered (assuming that the model is relevant for this kind of situation and partitioning of course).

This view fits well at least with the models of physics that are built on a state-space, or a history space as in Lagrangian mechanics. The identifying characteristics of the situation at hand determine values for model parameters: the strength of external potentials, initial conditions, etc. The partition of possibility space considered determines a partition of state-space (or history space). From this, a weighing can be inferred by projecting the model onto this partition. In the case of a deterministic model describing a trajectory in state-space, for instance, a cell of the partition can be considered possible only if the trajectory of the system intersects this cell, or it can be given a probability weight proportional to the time spent by the trajectory in the cell. So, technically speaking, the content of such models is indeed a function from context (parameter values and a partition of state-space) to contextual content (weighing of the cells of the partition).

Assuming this, the main question that ME attempts to answer is: when and how can we say that a theoretical model is *empirically adequate*?

It is important to remember that in the context of empiricism, empirical adequacy is an ideal notion (including in van Fraassen (1980)’s constructive empiricism). It does not express the fact that a theory or model is well confirmed by our observations so far, but rather that it would be confirmed by any observations we could possibly make.^4^ Empirical adequacy plays for empiricists the role that truth plays for realists: it is not identified with, but *inferred from* empirical success, in the minimal sense that empirical success gives us good reasons to believe that a theory is empirically adequate, and identified with an ideal aim for science to pursue.

We have already explained when an applied model is extensionnally accurate in context: when what it excludes does not actually happen. According to ME, a theoretical model is empirically adequate if it would be accurate in *all possible contexts*. This is a modal notion: adequate just means *necessarily accurate*, in an objective sense of necessity. Hence the “modal” in “modal empiricism”. The possible contexts considered include not only any perspective one could adopt on actual situations of the relevant kind (e.g. any conceivable partition of possibility space for objects that are actually dropped, up to a degree of precision for which the model is still valid), but also on counterfactual situations, which are alternative ways actual situations could be or could have evolved. So, for a model of free fall to be adequate, not only must it be the case that all dropped objects actually fall towards the ground, but also, it must be the case that they *could not* have gone up, and also that ceteris paribus, all objects *would* fall towards the ground if they were dropped, even if they were not, all this in virtue of natural constraints, holding fixed the broad environment of these situations. In other words, actual situations must fulfil the model’s conditions of accuracy as a matter of natural necessity.^5^

This notion of adequacy is empirical because it only concerns characteristics of the world that can be discriminated empirically, but the strict extensionality of traditional empiricism is not retained (explaining the motivations for this view is beyond the scope of this article: see Ruyant (2021, ch. 4.2, 5.2)).

### Modal epistemology (ch. 5)

Once this modal notion of empirical adequacy is in place, the central question is: how can we infer that a theoretical model is empirically adequate from its empirical success? ME’s answer is: by enumerative induction on contexts and situations of the same kind.

Enumerative induction is an inference by which a characteristic found in a sample of individuals deemed representative of a larger set is projected upon all individuals of this larger set. Assume that the realised situations we experiment upon are a representative sample of the larger set of all possible situations of the same kind (heavy objects being dropped or thrown). These are the actual situations of the same kind upon which we do *not* experiment (object dropped when we do not look), but also the alternative ways they could be, and the alternative ways situations on which we do experiment could be or could have evolved (what would have happened if non-dropped objects were dropped, etc.), all this taking into account the natural constraints on possibilities induced by the nature and environment of these situations. If something never happens in our accessible sample, then we can infer, by induction, that it never happens in the full set of possible situations of the same kind: it *cannot* happen for situations of this kind. This is just saying that if the model is accurate in our sample, then we can infer by induction that it is empirically adequate.

ME argues that this induction is fallible, but as good as any other kind of enumerative induction, at least once it is granted that possible situations exist (their existence should be motivated independently). Of course, we should make sure that our sample is representative of the set of all possibilities, by varying the circumstances of applications. This corresponds to what scientists do in experiments: they vary the value of parameters, the circumstances, etc., as if exploring possibility space, which plays in favour of this account (but again, a full defence of ME is beyond the scope of this article).

### Summary

Let us summarise the non-probabilistic version of ME’s modal epistemology, for the sake of a future comparison:

1. A context defines an a priori partition of coarse-grained possibilities for a situation with identifying (rigid) characteristics.
2. An applied model excludes some of these possibilities. The model is accurate if the possibilities that it excludes are not actually observed in context.
3. A theoretical model is a function from context to applied model. The model is empirically adequate if the possibilities that are excluded by the model are objectively impossible for all relevant situations, in all possible contexts (it reflects what is possible or not in general for situations of a given kind).^6^
4. We can infer by enumerative induction that a model is empirically adequate if it is accurate in a range of contexts that is representative of all possibilities.

## How to interpret probabilities?

So far, we have a picture of how scientific models represent possibilities, and how we can know by induction that they do so adequately. The problem with this picture is that it does not apply to probabilistic models. This inductive epistemology assumes that possibilities are either excluded or allowed by models, without any in-between. This is problematic, because the fact that many scientific models are probabilistic is one of the reasons to assume that there are possibilities in the world in the first place, and that we need a modal epistemology. Luckily, this account can be extended to the probabilistic case. In the next two sections, we first discuss the nature of the probabilities that scientific models describe, and then go on to provide an extension of Modal Empiricism that applies to them.

### Theories of probabilities

Let us review the options, following Suárez (2020). We can divide the interpretations of probability into i) subjective or epistemic, and ii) objective or ontic. Roughly (i) take it that probabilities are ‘in the head’ of the reflective agent or scientist; they are not part of the furniture of the world but feature only in our cognitive and representational processes and states. By contrast (ii) assume that at least some probabilities are “in the world”; they are features of the physical constitution of reality, whether it be as properties of statistical ensembles, or as dispositional properties of objects. The labels can be blurred, in that there are objective elements in some epistemic accounts of probability, such as objective Bayesianism, and vice versa: most ontic conceptions of probability assume that at least some judgements are required to shape up regularities into the right kind of probabilities. However, the distinction points to a clear ontological distinction regarding the sorts of systems that can be meaningfully said to exhibit or possess probabilities. In the subjective or epistemic conception, these are states of knowledge, information, or belief in the minds of agents. Objective conceptions take it that there are systems, states or processes in nature that exhibit such probabilities, regardless of any agents or their cognition.

The first alternative is the main contender for an epistemic conception of probability, namely subjective Bayesianism. In the wake of the so-called Ramsey-De Finetti theorem it is well-known that partial degrees of belief must be represented by probabilities if they are coherent. (Suárez, 2020, ch.3; see algo Gillies, 2000) This opens space for a thoroughly subjective interpretation of probability, where probabilities are just representations of ignorance, or incomplete information; they measure partial degrees of belief under conditions of limited information and instruct an ideal rational agent to adjust their degrees of belief in response to incoming evidence. Another contender, objective Bayesianism (Williamson, 2010), incorporates objective elements and is rather in the tradition of Keynes’ (1921) *principle of indifference*. Either of these proposals ensues in a roughly inductive calculus for inductive or probabilistic inference as a measure of evidence.

Since we are concerned with how scientific models represent real worldly possibilities, we shall put aside subjective and epistemic conceptions of probability. As was noted, we are concerned partly with the inductive weight of evidence for and against models, but we are also assuming outright that there are objective possibilities out there in the world, and that scientific models are in the business of describing and quantifying them by means of objective probabilities. Hence, we cast aside all attempts to understand probability entirely as a measure of evidence or ignorance.

There are two grand families of objective interpretations of probability, in terms of frequencies and propensities. The frequency and the propensity interpretations of probability thus each come in some variety. The former typically identify probabilities with frequencies of given attributes in some sequence of outcomes of a particular chance setup. The critical issues here concern the definitions of “frequency”, “attribute”, “sequence”, and “setup”, and different frequency interpretations give different accounts of these terms. On the simplest *actual frequency* account, any frequency of any given distinguishable attribute in the finite sequence generated by a stable chance setup that allows for repeatable trials of the same experiment provides an empirical probability of that attribute (in that sequence). Hard questions arise then regarding any putative extension of the same trial. Suppose we have tossed a coin 100 times and observed 50 heads and 50 tails precisely. This actual frequency in this finite sequence provides a probability for landing heads/tails for a toss of this coin. However, if we toss it another 100 times, we may not be able to preserve this exact ratio, and the question then arises whether we are compelled to change our probability ascription. The actual frequency account would seem to suggest so, but our intuition is clearly quite different – to stick to the first estimate. Any finite frequency can after all differ from the actual underlying probability and is in fact expected to do so within a range.

To overcome these difficulties appeals have been made to limiting or hypothetical frequencies. (See Hájek, 1997; 2009 for critical discussion). A limiting frequency is a mathematical abstraction – the number, if there is one, that the given frequency tends to in the limit when the sequence of outcomes goes to infinity. In the coin toss: the ratio of heads to tails that results when we let the sequence of tosses extend indefinitely. However, it is not always the case that this number exists. Many sequences diverge rather than converge in the limit. It is also strange (and would be contrary to the spirit of our proposal for probabilistic models anyway) to identify probabilities with abstract formal entities.

The alternative is to consider the hypothetical sequence that would result from tossing the coin indefinitely in the given circumstances, regardless of any mathematical limit of the set of intermediate finite sequences. However, note that any frequency ratios in such hypothetical sequences would themselves be hypothetical – i.e., they would incorporate the modality characteristic of a hypothesis regarding some possible development of a series that is not yet actual. In other words, hypothetical frequentism is not motivated by any sort of non-modal empiricism that would appeal only to the realm of the actual. On the contrary, it is fully committed to modality, and assumes that probabilities are modal properties or features of ideal sequences generated in hypothetical circumstances. The coin has in this actual world a probability ½ to fall heads only because an ideal sequence of tosses of that very coin in some possible world exhibits that frequency. This approach thus conveniently lines up with our account of modal epistemology for probabilistic models, which rejects the reduction of modal notions to non-modal ones. (As we have already pointed out, Ruyant’s (2021) ME is not a reductionist attempt to do away with modality. On the contrary it is an attempt to find room for objective modalities within a broadly pragmatist scientific methodology.)

We avoid hypothetical frequency interpretations nonetheless for two different reasons. First, the implicit appeal in these interpretations to possible worlds is incoherent with our rejection of possible world semantics for the sorts of situational possibilities that interest us. And there does not seem to be any other plausible semantics for hypothetical sequences, which are best understood as global descriptions of events through the history of some possible world that incorporates the actual world up to the present time. In other words, we cannot see how to apply our local and contextual situational semantics to the global hypothetical sequences that are demanded by hypothetical frequentism. Secondly, hypothetical frequencies are unsuited to our purposes, since they are group characteristics of a whole indefinite ideal sequence of events. That is, if probabilities are hypothetical frequencies, then they are properties of entire collectives or sequences of events. For our purposes we would like rather an account of probability that allows for single case chances; an account that allows us to ascribe objective probabilities to single events occurring to systems regarded in isolation (i.e., a single toss of a coin regarded in isolation), in the same way as the non-probabilistic version of ME attributes modal properties to single occurrent situations.

The propensity interpretation would seem to provide just this since contrary to what happens in a frequency account, it ascribes a property to the chance setup or system (or situation) that ought to be enough all by itself to yield the required probability for even just one operation of the chance setup. The idea is, roughly, that every time the chance setup operates in the actual world it displays the outcome of an underlying dispositional property with a given probability – a *bona fide* property of the one exercise of the setup. There is therefore no overt appeal, at least, to any global sequence of events, or collective, no implicit or explicit reference to global possible worlds, and chances are putatively understood fully to be properties of single events occurring to setups regarded in isolation.

However, there is discussion amongst defenders of propensities about how reliant propensities ultimately are on either global sequences, or “collectives” in Von Mises (1957) sense of the term. The long-run version of the propensity interpretation (Gillies, 2000) tries to stay resolutely empiricist (in the strict non-modal sense of empiricism) by conceptually connecting propensities to the long run sequences that chance setups generate. Thus, on this view, while the explanation still appeals to properties of the setup, it only appeals to those properties of the setup that are responsible for an indefinitely large sequence of events that broadly satisfy the conditions for a Von Mises collective. Putting aside issues regarding the definition of these collectives and whether they can be fully characterised in actualist terms, this again moves us away from our explicit aim to provide an account of probability that applies to the single case, i.e., that provides single case chances. The alternative is precisely the single case propensity accounts defended by Mellor (2005) and others, which explicitly make it clear that propensities are not in any way properties of collectives or sequences (actual or hypothetical) but properties of chance setups exercised in every instance of their operation. Thus, a single coin tossed only one time – and thereafter, say, destroyed – also possesses a propensity to land heads or tails, and it displays that propensity in the only one occasion in which it is tossed throughout its history, regardless of any collectives or sequences that this toss may be said to belong to in theory.

The single case propensity interpretation is thus roughly along the lines of what we call for. Yet it again has problems of its own, most notably so the so-called Humphreys’ paradox, which has been carefully described elsewhere (Humphreys, 1985; Gillies, 2000; Suárez, 2014). The upshot of this debate is that while propensities understood as dispositional properties of chance setups can yield or generate single case chances for events in those setups, *they cannot be identified with them*. Propensities and chances are not the same thing but are rather intricately related; propensities are those underlying properties of systems that can explain the single case chances that certain events within those systems display.

### The complex nexus of chance

We have seen so far that probabilities cannot be strictly identified with either propensities or frequencies, and yet, they seem related to both. Suárez (2020) has referred to this intricate link as the Complex Nexus of Chance (CNC). Most objective chances – not necessarily all – are explainable as the displays of underlying propensities, and frequencies of attributes in a sequence are themselves the manifestation of these chances. Yet, the chances displayed and the propensities that explain them are not the same kind of thing, nor are the chances identical to their manifested frequencies. Propensities are underlying dispositional properties of chance setups, while objective chances are the probabilities of certain outcomes displayed by events that are generated in those setups under specific circumstances. The former can be understood along the lines of several theories of dispositions, but no further assumptions shall be made regarding them here. The latter can be interpreted in a variety of different non-metaphysical ways, including Hoefer’s (2019) most recent best-system analysis (see Suárez, 2021 for a review of its most pragmatic components), but as a matter of fact do not require an interpretation (in line with Sober’s 2010 no-theory theory of probability, endorsed in Suárez, 2020, Ch. 10).

This three-tier distinction between propensities, single-case chances and frequencies has strong affinities with ME. Recall that according to ME, theoretical models are functions from context to applied models: theoretical statistical models would therefore represent the dispositional properties of objects or kinds of objects, while applied statistical models would represent their single chances in a particular context. Accordingly, the adequacy of theoretical models, in a probabilistic version of ME, should correspond to the fact that they “get probabilities right” in all possible contexts (which is the analogue of being correct regarding what is objectively possible or impossible in deterministic models). In other words, this is fulfilling the requirement in Suárez (2020, p. 59) that “propensities (understood as dispositional properties of chance setups) are not tested against finite frequency data, but against probability distributions within statistical models of the phenomena”. We are thus implicitly providing a way to test abstract propensity claims against probability models grounded on frequency data, in agreement with a tripartite conception of objective chance such as the one in the CNC.^7^ This adequacy should be inferred from the extensional accuracy of concrete applications, where frequencies would play a central role. Our goal, in the next section, is to fill-in the details of this rough picture.

## Probabilistic modal empiricism

Having settled on an objective chance interpretation of probabilities, we can now examine how the modal epistemology presented in Sect. 2 could be extended to account for probabilistic models. We shall assume, in line with ME, that a theoretical model is a function from context to applied models, where the context corresponds to a specific target system to which the model is applied, associated with specific values for dynamical parameters and a partition of possibility space that typically corresponds to our empirical discrimination abilities. As already explained, the probabilities are then assumed to appear in the applied model (but not necessarily directly in the theoretical model): they weigh the possibility space of the target situation and correspond to the objective chances of this situation. They are, as per CNC, the manifestations of propensities in particular contexts. The objective of this section is to extend the induction-based epistemology presented before to this picture.

### An issue with probabilistic adequacy

Let us start by noting that an extension of ME to the probabilistic case is not as straightforward as it could seem.

The problem is the following. Imagine we have a model of coin toss that tells us that the probability of heads is half. We toss a hundred coins and obtain roughly half heads and half tails. We would like to say that in this case, our observations confirm the adequacy of the model. However, the inductive account laid out previously does not fit well with this inference. Remember that according to this account, if something is excluded in all observed cases, then you can infer by enumerative induction that it is excluded in all possible cases. But the coin model *does not exclude anything*: it tells us that both heads and tails are possible outcomes for every toss.

In the non-probabilistic case, modal knowledge is acquired by extrapolating from the fact that an individual characteristic is present in situations of a sample to the conclusion that it is present in all situations in a larger set. This is an inference from individual characteristics to individual characteristics, which is just what enumerative induction is. But what we do in the probabilistic case is prima facie different: it apparently amounts to *inferring individual characteristics from a group characteristic* (single-case probabilities from a statistical distribution). This blocks a naive transposition of ME to the probabilistic case, or any account based on enumerative induction more generally. There is a non-homogeneity between the premise and conclusion of the inference, which means that the inference is ampliative in a stronger sense than enumerative induction: it is more of an *inference to the best explanation*. In the case at hand, we could say that probabilities *explain* statistical distributions, but the explanatory link between the two is not necessarily clear. In any case, this blocks a direct transposition of ME to the probabilistic case.

A way to resolve this issue is to conceive of a probability as a kind of modal frequency: a ratio among possibilities. This way, it would seem, the premise and conclusion of the inference are of the same nature: from the fact that about half of realised coin tosses give us heads, we infer that half of possible coin tosses would give us heads. More precisely, we could understand accuracy for an applied probabilistic model in terms of a fit between its probabilities and the frequencies displayed in a collection of events (which would then be a matter of degree), and the adequacy of the corresponding theoretical model in terms of accuracy in all or most collections of possible events. If an account of this kind were fully developed, then plausibly, the corresponding notion of adequacy would be justified by enumerative induction from accuracy, just as ME has it. Perhaps this could be made to fit with either a long-run propensities theory or a hypothetical frequencies theory of probabilities. However, as already explained, such theories do not fit very well with ME’s focus on situations, since it would appear that probabilistic models have as target systems collections of situations instead of situations. While leaving open the possibility of developing this option, we will focus here on the development of an epistemology that stays closer to non-probabilistic ME.

### A look at scientific practice

It could seem that the probabilistic case is not congruent with ME. However, having a closer look at how probabilistic models are tested in science does not warrant the idea that probabilities are directly inferred from statistical distributions (indeed, if they were, we would never infer non-rational probabilities). It actually gives us a picture that is very close to what ME suggests in the non-probabilistic case.

Take, for example, how Mayo and Spanos (2006) characterises a severe test:

Data *x*_*0*_ in test *T* provide good evidence for inferring *H* (just) to the extent that *H* passes severely with *x*_*0*_, i.e., to the extent that *H* would (very probably) not have survived the test so well were *H* false.

The idea is roughly that considering *H* to be false gives a very low probability, lower than a significance threshold, for what is actually observed in a test. Considering *H* to be false amounts to considering a null-hypothesis that is the disjunction of all alternatives to *H* to be true (within a set of relevant alternatives given by the research context). The test is severe if it allows for an outcome that is given very low probability by this null-hypothesis, but not by *H*. Considering models instead of hypotheses, assuming a set of relevant models that apply to the same kinds of contexts (in the sense of ME), the narrative can be roughly paraphrased in this way:

1. A model is discarded when what it gives very low probability to is actually observed.
2. A model passes a severe test when all alternatives but the model are discarded.^8^

A severe test must allow us to discriminate between the model and all relevant alternatives, by allowing for an outcome that would discard all but the model.

Mayo and Spanos are interested in p-value testing for hypotheses here, but this kind of narrative, thus paraphrased, can easily be generalised to the confirmation of models in general. The main idea is that accepting a model or hypothesis requires discarding all its alternatives, which means exploring the space of possibilities (see Schupbach (2018) for an account of robustness along these lines), eliminating potential sources of errors, etc. And this is roughly what ME says. Point (1) above is almost exactly what our point (2) at the end of Sect. 2 states, namely that a model is discarded (not accurate) if what it excludes is actually observed. We simply need to assimilate low probability attribution with exclusion of a possibility. As for (2), it corresponds closely to point (4) from Sect. 2, which says that we can infer adequacy from accuracy in a representative range of contexts. As explained previously, the assumption that our sample of situations is representative involves making tests in a large enough range of varying circumstances, which means, in effect, confronting the model to all relevant alternatives, at least assuming that these alternatives would make a difference in at least one possible context: in at least one context, they attribute very low probabilities to an outcome while our confirmed model does not.

However, what is important to understand is that the models that are directly put to the test are *not* in general basic probabilistic models. They are, more typically, composite models representing *sequences of independent events*, and attributing probabilities close to 1 and 0 of getting frequencies inside or outside a range. As a matter of fact, the frequencies of outcomes that are attributed high probabilities will correspond closely to the probabilities of the basic model, thanks to the central limit theorem. Considering, for example, a model of 100 independent coin toss, where each toss has a half probability of yielding heads or tails, would give a very high probability of obtaining roughly as many heads as tails, between 45 and 55, say. But what is tested is not the basic coin toss model: it is a composite model, constructed from this basic model and other assumptions (the statistical independence of every toss), with the notable feature that it yields all-or-nothing probabilities for specific outcomes (having frequencies inside or outside a range). And this is precisely the fact that such models yield “dichotomous claims” that makes them suitable for an empirical test, because these claims can be directly compared to what is observed and yield a determinate conclusion, e.g. that such alternative model is discarded (Tunç et al., 2023).

The lesson from these observations (which we do not take to be necessarily novel) is this: mild probabilities are never inferred directly from frequencies of outcomes. Only all-or-nothing probabilities (1 or 0), corresponding to necessities and impossibilities, are ever confirmed experimentally. However, such probabilities can act as proxies for confirming models with mild probabilities, with the mediation of composite models, such as models of sequences of independent events.

### Probabilistic empirical adequacy

This analysis points to a solution to our problem. Firstly, we can keep ME’s conception of empirical adequacy for the models that are directly confronted with experiments, by simply replacing the “excluding” in the original version by “giving a low enough probability weight”. Secondly, this picture can be completed with an account of how to infer, from the adequacy of a composite model constructed from more basic models, that the basic models are also empirically adequate.

“Giving a low enough probability weight” is not very precise, but this is not necessarily problematic. What counts as empirical success can vary from one context to the other, in particular regarding the risk of error that one is willing to take. So, we can introduce a threshold γ, specified by the context, and assume that a model is accurate in context if the possibilities that are attributed a weight lower than γ do not actually occur (taking γ < 1/*N* for *N* possibilities, to avoid cases where accuracy is impossible to achieve). The model will be discarded if these possibilities occur, and we would typically want γ to be small if the test is severe, because we don’t want to discard alternative models too easily. However, this would ultimately depend on the context of application.^9^

But there is another issue that should be addressed. In the non-probabilistic case, empirical adequacy just is accuracy in all possible contexts. This won’t do for the probabilistic case: we cannot infer, from empirical success, that a model would be accurate in *all* possible contexts. For example, a model of 100 coin tosses would be accurate in all possible contexts if necessarily, such sequences of tosses could only yield between 45 and 55 heads (assuming a particular threshold), but this is not correct: what the model actually says is that there is a small probability of getting a result outside of this range. The solution to this problem is to claim that an adequate model is accurate in *most*, not *all* possible contexts, and the natural way of interpreting this is to say that its probability of accuracy is high enough, or that for all situations, the objective probability to display a possible outcome that is given a very low probability by the model is equally low (lower than a threshold which could be made to contextually depend on γ above).

We would not necessarily want to *identify* adequacy with probable accuracy in all possible contexts, however. A slightly stronger claim is that the probabilities attributed by the model to various measurable possibilities correspond to objective chances. The weaker claim is a consequence of this stronger claim: if the model “gets probabilities right”, then its probability of inaccuracy (which is the probability of getting a result to which it attributes probabilities below γ) is low (it is lower than *n∙*γ, where *n* is the number of possibilities with attributed probabilities lower than γ). Whether this stronger claim is an adequate characterisation of empirical adequacy, that is, what would make a model ideally acceptable for an empiricist, depends on whether it can be inferred by induction from empirical success. And this is where the clause on composite models intervenes.

The reasoning is roughly the one from Mayo and Spanos in the citation above. If our model did not get probabilities right (if it were “false”), then there is at least one context involving a composite model constructed from it where this composite model would not be accurate with very high probability (it would “very probably not have survived the test so well”). Scientists can actively create a variety of contexts and composite models to test for this. If the composite models are accurate in all such contexts, then we can infer that our original model gets probabilities right, at least in approximation, because all alternative models yielding different composite models are discarded up to a level of precision. Although it could be said that the probabilities of the basic model explain, in some sense, the statistics predicted by composite models, this explanatory power is more a consequence of empirical adequacy than a way of justifying the model: inferring that the basic model gets probabilities right is *not* an inference to the best explanation, at least not in the sense that some non-empirical virtues (simplicity etc.) would be invoked on top of empirical success. The point is rather that the accuracy of composite models in very specific situations informs us that some of the probability assignments that can be derived from the basic model are roughly correct (the ones that are very low), and by induction on various model combinations, by implementing a variety of different situations, we can infer that all probability assignments are roughly correct, and with more and more precision.

### Summary

To sum up,

1. A context defines an apriori partition of *N* coarse-grained possibilities for a situation with identifying characteristics, and a risk threshold γ < 1/*N*.
2. An applied model attributes probabilities to these contextual possibilities. The model is *accurate* if the possibilities that are attributed a probability lower than γ are not actually observed in context.
3. A theoretical model is a function from context to applied model. It is empirically adequate if the probabilities assigned to contextual possibilities correspond to the objective probabilities of these possibilities obtaining.
4. We can infer that a model is empirically adequate if
  - it is mostly accurate in a large representative range of contexts
  - composite models constructed from it (using the compositional rules of the theory and possibly other adequate models) are also accurate in a representative range of contexts.

The main changes from our non-probabilistic version are that (i) we added a threshold γ to the context, (ii) we replaced “excluding” with “attributing a probability lower than γ”, (iii) we replaced “the possibilities that are excluded by the model are objectively impossible” with the idea of attributing the right probabilities (which implies that the probabilities given very low weight would normally not be observed), and (iv) point 4 now incorporates the idea of using model composition in order to fully test a model (something already alluded to in Ruyant (2021, ch. 3.3.5, p. 87)). This shows how propensities, conceived of as functions from context to probabilities, can be known by induction on experience.

## Concluding remarks: is it still empiricism?

Modal Empiricism proposes that we can know about what is possible or not by induction on situations, assuming that actual situations of a type are representative of possible ones. This modal epistemology can be extended to the probabilistic case. Extreme probabilities, close to 1 and 0, are tested empirically in the same way as necessities and impossibilities in the non-probabilistic version of ME. Mild probabilities are assessed indirectly, through the construction of composite models, such as models of sequences, that “convert” them into high or low probabilities of frequencies.

This idea that models should be considered adequate not only when they are directly confronted with experimental data, but also when they can successfully be combined into more complex models that are empirically successful, was already hinted at in (Ruyant, 2021, Sect. 3.3.5) but it becomes much more central in a probabilistic extension of ME. This brings into ME a coherentist component that could play a role in accounting for how probabilities can be assigned to single events that cannot be reproduced into sequences (for example the probabilities of different scenario in climate science). We cannot expect the probabilities that figure in such complex models to be “severely tested” in the way explained in the previous section, but when complex models are built from simple ones that are empirically adequate, this gives us reasons to trust that they are themselves at least approximately adequate: the probabilities that figure in them are good guesses about objective likelihoods. This means that the present epistemology is flexible enough to account for a variety of uses of probabilities in science.

What is markedly inductive in this account (and goes beyond the hypothetico-deductivism of Mayo and Spanos from which it is inspired) is that we allow ourselves to infer, from the fact that a model passes a variety of severe tests, that it would pass any possible test, and therefore that it “gets probabilities right”. Of course, such inference is defeasible, as is the assumption that our sample of tested circumstances is representative of all possibilities and that all relevant alternatives were explored. But this was already the case with non-probabilistic modal empiricism. Having said that, one could worry, in relation to probabilistic ME specifically, that introducing model composition opens an unfathomable range of possibilities, and that pragmatic or theoretical limitations become necessary to make this possibility space manageable. This would mean that the account cannot be purely empiricist. Perhaps a priori theoretical assumptions are also involved in establishing acceptable model composition rules. Finally, another place where extra-empirical considerations could intervene is that we cannot expect adequate models to be *necessarily* accurate, for reasons already given: rather they are accurate with high probability, and the tolerance to inaccuracy can be a matter of evaluating the risks involved in context. Concerning this latter aspect, let us note that the inductive process that we think is involved in assessing whether a model is adequate (which must involve a “severe” test) is likely to wash out all contextual aspects, including any use of contextual values during concrete applications, because the aim, according to probabilistic ME, is to have models that are likely to be accurate in *all* contexts, whatever the tradeof between false positive and false negative that one is willing to adopt in context. This simply means models that “get probabilities right”, which does not depend on contextual values. As for the other aspects, we take a pragmatic attitude to the theoretical choices that must be made. That is, we assume that these choices answer not to assessments of fit with reality, or any other metaphysical category, but rather strictly to reasons of pragmatic utility and plausibility within contexts of inquiry of the sort endorsed by (Suárez, 2020) for statistical modelling methodologies. In addition to hypothetico-deductivism and strict inductivism, there are a variety of abductive features to those methodologies. Whether our adopting these features is enough to claim that we have left the territory of strict empiricism in favour of a pragmatist account, or something else, remains to be examined,^10^ but whatever the answer, we do not think that this undermines the account.

## Data Availability

N/A.

## Footnotes

1 ME as a position in philosophy of science must be distinguished from modal empiricism in metaphysics, which opposes modal rationalism (see Fischer and Leon 2017). There are affinities between the two approaches, notably the idea that modal knowledge can be acquired by induction on experience, as well as a particular focus on *de re* possibilities (possibilities *for* an object or situation rather than possibilities in general) (see Roca-Royes 2023). However, the two are different. ME in philosophy of science is a position about the significance of scientific theories. It takes place in the context of the debate on scientific realism, where it constitutes a middle way between non-modal empiricism and full blown scientific realism, and it is concerned with natural or nomological possibilities, not with metaphysical possibilities. The label has been used in this sense by Giere (in Churchland and Hooker 1985, ch. 4) and by Ladyman and Ross (2007).

2 This analysis in terms of partition of possibility space is similar to accounts of “aboutness” that have been proposed in philosophy of language, drawing from Lewis (1988): see Yablo (2014); Hawke (2018); Berto (2022)

3 This notion of accuracy is weaker than the one usually entertained in formal epistemology (e.g. Pettigrew 2016). It roughly corresponds to the applied model not being falsified by observations.

4 Van Fraassen has a particular interpretation of this modal that avoids any commitment to objective modalities, but this is still an appropriate way of characterising his view (see Monton and van Fraassen 2003).

5 This idea is based on an account of possibilities that is not exactly the possible worlds semantics. It conceives of possible situations as centred on (or accessible from) actual situations with rigid identifying characteristics, so that the necessity operator □_s_ is indexed to a situation of reference. Empirical adequacy is roughly a necessity claim of the form (∀s)(□_s_ As) (Ruyant 2021 ch. 4.2.6, 5.2). But the details do not matter for our purpose.

6 A caveat is that in this picture, a model that is not completely informative because it does not exclude all impossibilities can still be empirically adequate. This caveat will disappear in the probabilistic case.

7 For the abstract nature of propensities, see Suárez (2025)

8 This is not exactly what Mayo says, because the disjunction of all these models should be discarded rather than each of them independently, but this is close enough for our purpose.

9 The risk of error that one is willing to take in a context presumably depends on a value judgment, where the values involved could be either epistemic, pragmatic or socio-cultural (as per the inductive risk argument). We do not wish to take a stance here regarding which of these kinds of values have a legitimate role to play in scientific practice: our approach is compatible with any stance on this question.

10 Ruyant (2021, ch. 6–7)’s strategy to address problems of theory change, which amounts to assuming that empirical adequacy is always relative to an unspecified background context, could be coopted here.
